# High-Resolution Intravital Microscopy

**DOI:** 10.1371/journal.pone.0050915

**Published:** 2012-12-14

**Authors:** Volker Andresen, Karolin Pollok, Jan-Leo Rinnenthal, Laura Oehme, Robert Günther, Heinrich Spiecker, Helena Radbruch, Jenny Gerhard, Anje Sporbert, Zoltan Cseresnyes, Anja E. Hauser, Raluca Niesner

**Affiliations:** 1 German Rheumatism Research Center, a Leibniz Institute, Berlin, Germany; 2 LaVision Biotec GmbH, Bielefeld, Germany; 3 Charité – University of Medicine, Berlin, Germany; 4 Max-Delbrück Center for Molecular Medicine, Berlin, Germany; University Hospital Jena, Germany

## Abstract

Cellular communication constitutes a fundamental mechanism of life, for instance by permitting transfer of information through synapses in the nervous system and by leading to activation of cells during the course of immune responses. Monitoring cell-cell interactions within living adult organisms is crucial in order to draw conclusions on their behavior with respect to the fate of cells, tissues and organs. Until now, there is no technology available that enables dynamic imaging deep within the tissue of living adult organisms at sub-cellular resolution, i.e. detection at the level of few protein molecules. Here we present a novel approach called multi-beam striped-illumination which applies for the first time the principle and advantages of structured-illumination, spatial modulation of the excitation pattern, to laser-scanning-microscopy. We use this approach in two-photon-microscopy - the most adequate optical deep-tissue imaging-technique. As compared to standard two-photon-microscopy, it achieves significant contrast enhancement and up to 3-fold improved axial resolution (optical sectioning) while photobleaching, photodamage and acquisition speed are similar. Its imaging depth is comparable to multifocal two-photon-microscopy and only slightly less than in standard single-beam two-photon-microscopy. Precisely, our studies within mouse lymph nodes demonstrated 216% improved axial and 23% improved lateral resolutions at a depth of 80 µm below the surface. Thus, we are for the first time able to visualize the dynamic interactions between B cells and immune complex deposits on follicular dendritic cells within germinal centers (GCs) of live mice. These interactions play a decisive role in the process of clonal selection, leading to affinity maturation of the humoral immune response. This novel high-resolution intravital microscopy method has a huge potential for numerous applications in neurosciences, immunology, cancer research and developmental biology. Moreover, our striped-illumination approach is able to improve the resolution of any laser-scanning-microscope, including confocal microscopes, by simply choosing an appropriate detector.

## Introduction

Since its development in 1990 [1], two-photon laser scanning microscopy (TPLSM) significantly contributed to a better understanding of cellular dynamics in adult small animals. For instance, TPLSM has been applied to analyze immune responses *in vivo* and to understand pathophysiological processes in the central nervous system [2]–[7]. Due to the advantages of near-infrared short-pulsed two-photon excitation [8]–[9], TPLSM for the first time allowed cellular and subcellular dynamic deep-tissue imaging in vital organ models and, even more important, in organs of anesthetized animals, i.e. intravital imaging [4], [10]–[12].

Although standard TPLSM was able to answer many questions in biosciences, there are still technical limitations with regard to imaging depth, deep-tissue spatial resolution and photobleaching/phototoxicity [13]. In particular, these limitations are obvious in the compact tissue of adult small animals, which is prone to scattering of both excitation and emission radiation, resulting in a depth-dependent deterioration of spatial resolution in tissue [14]–[15] due to spherical aberrations of the point spread function (PSF) [16]. This prevents us from identifying dynamic cellular interactions deep in living organisms, which build a central and general mechanism of tissue and organ function, e.g. in the neuronal synapse, the immune synapse (kynapse) [17] or in the neuro-immune synapse [6]. Using standard TPLSM, fine structures like processes of cells cannot be detected and there is a high risk of identifying false contacts due to deep-tissue PSF aberrations.

To unequivocally visualize these cellular contacts and their dynamics, a highly improved spatial resolution is needed deep within tissue. Various fluorescence nanoscopy (super-resolution) techniques improve spatial resolution well beyond the diffraction limit. STED/RESOLFT [18]–[20], pattern-illumination and standing-wave techniques [21]–[24] as well as single-molecule localization techniques like PALM, STORM, dSTORM [25]–[27] have been developed and applied to achieve super-resolution images of fixed samples and live cells in culture.

Two-photon excitation STED microscopy has been demonstrated [28] and applied to deep-tissue imaging in brain slices [29] and to image microtubuli within cells [30], resulting in an improvement of lateral resolution – 280 nm in 130 µm depth in brain slices and 85 nm in single cells. One-photon excitation STED was recently employed to dynamically image dendritic spines at the surface of mouse cortex up to 10–15 µm depth *in vivo* [31], reaching a resolution of 67 nm. Multifocal structured-illumination microscopy was used to double lateral resolution in translucent organisms like zebra fish or mouse embryos [32] but not in the highly light-scattering tissue of adult animals.

The diffraction theory predicts a 3-fold worse axial resolution as compared to the lateral resolution [15]. In deep-tissue imaging both lateral and axial resolution are affected by scattering effects to a similar extent [8]–[9], [15]. Together these two aspects make axial resolution the limiting factor at a large imaging depth. However, the above-mentioned techniques focus on breaking the diffraction limit and on improving especially lateral resolution. Moreover, the existing super-resolution techniques are still mostly incompatible with dynamic deep-tissue imaging (i.e. several hundred µm) due to (i) the use of one-photon excitation, which limits the penetration depth in tissue (except for two-photon excitation STED), (ii) incompatibility of the set-up with large samples like whole organs (in the case of single-molecule localization techniques), (iii) incompatibility with optical non-linear signals such as second harmonic generation (SHG) or third harmonic generation (THG), (iv) photobleaching and photodamage effects and (v) spheric aberrations of the wave front due to refractive index mismatches in deep tissue resulting in incapability to dynamically image large 3D areas (e.g. 300×300×50 µm^3^) as required by typical biological questions.

Despite the possibility to employ a blind estimation of the point spread function (PSF), for instance using Huygens© software, the improvement of resolution based on post-evaluation, i.e. image 3D-deconvolution, is limited due to the complex PSF-dependence on both cellular composition and imaging depth in tissue [14]. In this respect, pre-acquisition correction of PSF-aberrations using adaptive optics is a better choice [33]–[34]. However, since the refractive index distribution in tissue is heterogeneous and can vary on the scale of nanometers, a time-consuming pixel-by-pixel PSF-correction would be necessary, thus slowing down the acquisition.

Approaches implying temporal or spatial modulation of the excitation pattern, i.e. SPADE (structured pattern applied to detection) and SPIN (structured pattern illumination) have been theoretically proposed to improve resolution in wide-field and laser-scanning microscopy [35], yet they haven't been developed further until now.

Therefore, a method which improves lateral and axial resolution deep within the organs of living adult animals and which can be used for time-lapse 3D imaging over several hours is still missing.

Here we propose striped-illumination multi-beam two-photon laser-scanning microscopy (MB-SI-TPLSM), as a method to improve axial and lateral resolution and contrast of both fluorescence and other optical non-linear signals (SHG, THG) by spatially modulating the excitation during the laser scanning process. To our knowledge, this is the first technique able to significantly improve axial resolution deep within highly-scattering tissue. It is therefore applicable to intravital imaging in various organs of adult small animals. The power of MB-SI-TPLSM is demonstrated by intravital imaging of cellular structures and communication in lymph node, spleen and brain as compared to standard TPLSM, i.e. single-beam-scanning photomultiplier-based TPLSM (SB-PMT-TPLSM) and multi-beam-scanning CCD camera-based TPLSM (MB-CCD-TPLSM). Furthermore, due to fast acquisition by MB-SI-TPLSM we are now able to dynamically quantify the interactions between immune complex deposits on the membrane of follicular dendritic cells (FDC) and B cells in germinal centers within the popliteal lymph nodes of live mice.

## Materials and Methods

### Setup for TPLSM

Experiments were performed using a specialized multi-beam two-photon laser-scanning microscope based on the scan head TriMScope (LaVision BioTec, Bielefeld, Germany). In brief, the beam of a tuneable fs-pulsed Ti:Sa laser (wavelength range 690–1080 nm, 140 fs, 80 MHz, Chameleon Ultra II, Coherent, Germany) is splitted into 2, 4, 8, up to 64 beams, forming a beamlet line. Two consecutive beamlets within the line have perpendicular polarisations and are shifted in time in order to avoid interference. The time-shift between two consecutive beamlets amounts to 3 ps. For image acquisition, the beamlet line is focused into the sample by an objective lens (either a 20× lens, NA 0.95, WD 2 mm – Olympus, Hamburg, Germany or a 40× lens, NA 1.1, WD 624 µm, Zeiss, Jena, Germany, both water immersion objectives) and perpendicularly scanned, so that a well defined periodical pattern is generated. This pattern is translated along the beamlet line. The series of images generated this way are detected through interference filters mounted on a filter wheel either by an EM-CCD camera (Hamamatsu C9100, Germany) or by a CCD camera (QE Sensicam, PCO, UK) and finally evaluated by customized algorithms (*Supplemental Material S1*). Single-beam TPLSM based on PMT-detection was performed with the same microscope. In this case, we used only one laser beam to scan the sample, and we spectrally resolved the fluorescence signal with corresponding dichroic mirrors and interference filters before detecting it with photomultiplier tubes (Hamamatsu HS4 and PMT, Germany).

### Mice and sample preparation for imaging

Detailed information about mouse handling and sample preparation for imaging can be found in *Supplemental Material S1*. The animal experiments were approved by the appropriate state committees for animal welfare (LAGeSo, Landesamt für Gesundheit und Soziales, Berlin) and were performed in accordance with current guidelines and regulations (animal experiment license G0153/08).

## Results

### Multi-beam striped-illumination two-photon laser scanning microscopy

**Figure 1:**
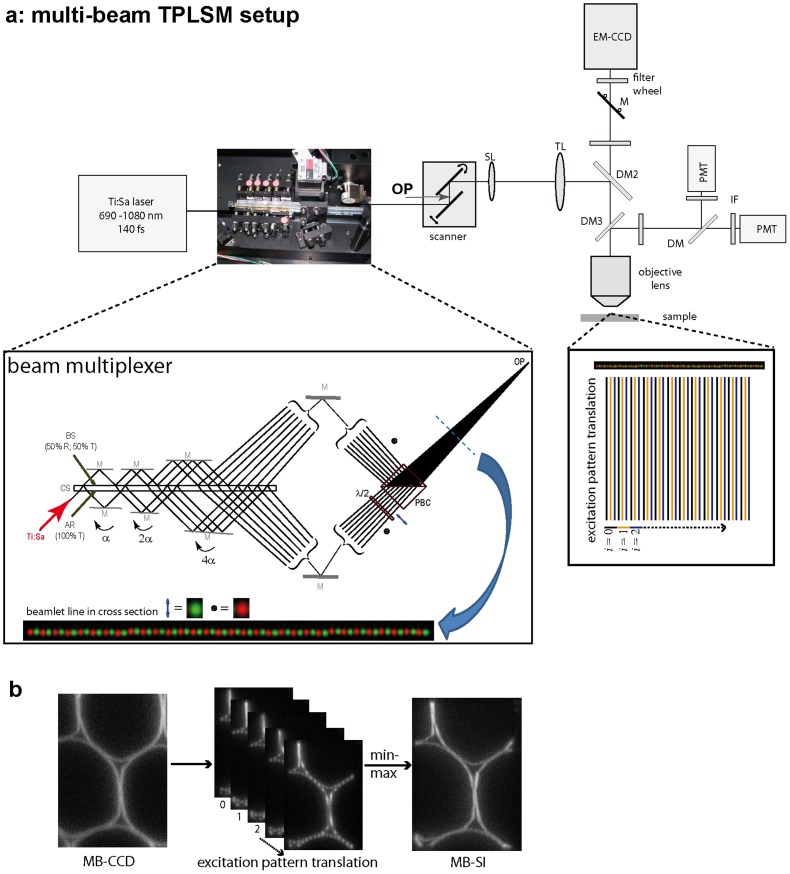
Principle of multi-beam striped-illumination two-photon laser-scanning microscopy. (**a**) The Beam-Multiplexer splits up the incoming Ti:Sa laser beam into up to 64 beamlets (only 16 are shown here for better visibility). It mainly consists of the central substrate (CS), high reflecting mirrors (M) on both sides and a polarizing beam splitter cube (PBC). Due to the use of only flat optics (λ/10 flat mirrors), aberrations are avoided. The incoming beam first enters the CS, where it is split into two beams of identical intensity (50% reflection/50% transmission) at the coated surface BS (beam-splitting). Both transmission and reflection at the BS surface oscillate only slightly around 50% if changing the laser wavelength, thus allowing for equal intensity splitting between the beamlets over a wide wavelength range (710–1060 nm). Each pair of highly reflective mirrors positioned on both sides of the CS directs the beams back onto the BS surface, where they are split into two again. After passing six pairs of mirrors (M), two sets of 32 beams emerge on each side of the CS. The polarization of one set is switched from perpendicular (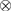) to parallel (↕) using a λ/2 plate before both sets are directed onto the PBC to be recombined in a comb-like structure. Thus, neighboring foci have opposite polarisation (for better understanding, the perpendicular-polarized (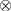) beamlets within the beamlet line are shown in red and the parallel-polarised (↕) ones are depicted in green). The whole arrangement forms a two-dimensional convergent beam fan with all beams having slightly different angles with respect to each other. The convergence is achieved by tilting the mirrors (M) on one side of the CS under different angles as indicated in the figure. On the other side of the CS, all mirrors are parallel to the CS. All beamlets superpose at the point of overlap (OP) that is located exactly between the mirrors of the two-axis galvanometric scanning system. As the objective lens transforms angles into lateral distances, a line of foci is generated at the object plane. The angle between neighboring beams amounts to 0.6 mrad leading to a distance of 30 µm for the corresponding foci at the focal plane of the scan lens. When using a 20× magnification objective lens, the distance of neighboring foci in the object space amounts to 1.5 µm. This spacing will be different when using another objective lens. (b) Working principle of “striped-illumination” exemplified on stained *Convallaria* roots: the beam line of multiples of 32 beam lets (one polarization) is perpendicularly scanned over the sample. This scanning pattern is translated in defined steps along the beam line, so that a series of images with different excitation patterns is generated. After evaluation with the minimum-maximum-algorithm, this image series results in an image of better resolution and higher contrast.

The periodical pattern in MB-SI-TPLSM is created by scanning multiples of 32 parallel beamlets of the same polarization in one direction, perpendicular to the beamlet line (Fig. 1a). This grid is translated along the multi beam line in steps *h* under the resolution limit over a distance *n·h*, which slightly overlaps the distance between two consecutive foci, i.e. 2.8 µm (112 steps of the galvo-scanner) to generate the spatial modulation of the excitation. The evaluation of the series of superposition images can be performed either with a minimum-maximum (MMA, similar to the algorithm used in HiLo microscopy), or with a Fourier-transform algorithm (FTA, similar to the one used in structured-illumination); details can be found in *Supplemental Material S1*. Thus, MB-SI-TPLSM is a further advancement of multi-beam (multi-foci) laser-scanning microscopy technique, which uses the spatial modulation of the scanning pattern to enhance contrast and resolution.

In contrast to HiLo and structured-illumination techniques – which are classical wide-field imaging techniques and achieve spatial modulation of the excitation by modifying the laser beam cross-section, either by moving a grid through the beam path or by bringing two or three beams to interference-, our technique uses only the laser scanning process without modifying the beam profile itself. Thus, our technique is a laser-scanning microscopy technique, perfectly compatible with two-photon excitation and, therefore with intravital deep-tissue imaging.

As expected, FTA allows a significant improvement of lateral resolution superior to MMA (*Supplemental Material S1*, *Fig. S1*). By means of MMA the axial resolution was explicitly improved. Only marginal enhancement of lateral resolution is achieved owing to reduced axial projections originating from out-of-focus regions. However, FTA is expected to be more prone to unpredictable sample movement, which is inevitable when imaging living organisms due to shift-artifacts of the translation components in the Fourier space. We therefore used the more robust MMA in all following experiments.

Optimal spatial resolution and adequate imaging speed is achieved for *h* between 175 nm and 250 nm (7 to 10 steps) as determined by varying *n*, *h* and *n·h*. The translation distance was 2.8 µm (112 galvanometer steps) for *h* = 7 and 3.75 µm (150 galvanometer steps) for *h* = 10, i.e. 15 frames. These values were determined using the 20× objective lens (NA = 0.95), for which the pixel size at the sample was 129 nm.

**Figure 2:**
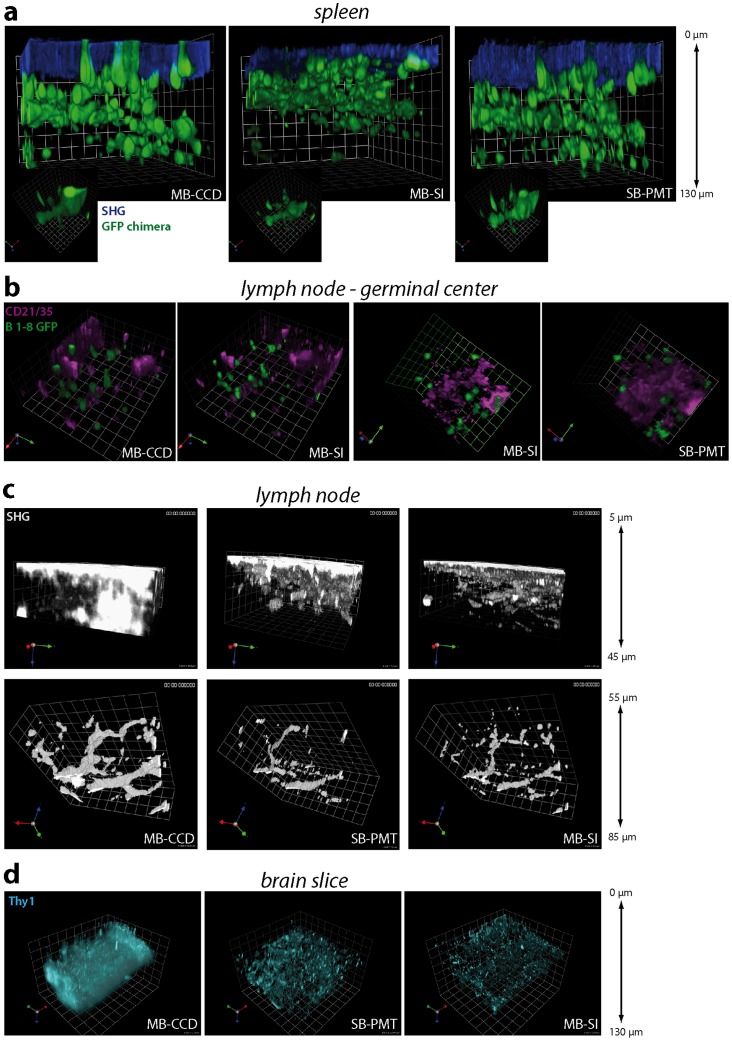
Comparison of multi-beam striped-illumination TPLSM to standard single- and multi-beam TPLSM. (**a**) 3D fluorescence images of spleen slices of chimera EGFP mice reconstituted with a non-fluorescent immune system using single-beam scanning PMT-TPLSM (SB-PMT), multi-beam scanning CCD-TPLM (MB-CCD) and SI-TPLSM (MB-SI). λ_exc_ = 920 nm, grid unit = 16.5 µm, grid unit of the cropped image = 2.0 µm. (**b**) 3D fluorescence images of the same regions within a popliteal lymph node after immunization with NP-CGG as recorded by MB-CCD-TPLSM versus MB-SI-TPLSM or by SB-PMT-TPLSM versus MB-SI-TPLSM. Follicular dendritic cells (FDC) are stained by CD21/CD35-Fab fragment-ATTO590 (magenta), while antigen-specific B1–8 cells express EGFP (green). λ_exc_ = 860 nm, grid unit = 15 µm. (**c**) 3D second-harmonic generation (SHG) signal images of the same region within a non-fluorescent lymph node as recorded by MB-CCD-, SB-PMT- and MB-SI-TPLSM. The SHG signal mainly originates from collagen fibers. λ_exc_ = 900 nm, grid unit = 10 µm (grid unit = 8 µm for MB-CCD-, MB-SI-TPLSM and 6 µm for SB-PMT-TPLSM). (**d**) 3D fluorescence images of acute cerebellum slices of CerTN L15 mice (expresses Cerulean and Citrine over the Thy1 cassette) recorded with the same set-ups. λ_exc_ = 850 nm, grid unit = 20 µm. All experiments were performed with the 20×, NA = 0.95 objective lens at *z*-step = 500 nm.

We expected MB-SI-TPLSM to have a crucial advantage in terms of resolution and contrast over standard TPLSM techniques in thick tissue, i.e. multi-beam CCD-based TPLSM (MB-CCD-TPLSM, adequate for fast imaging) and single-beam PMT-based TPLSM (SB-PMT-TPLSM, adequate for deep-tissue imaging). Therefore, comparative imaging experiments were performed to verify the applicability in different tissue samples: lymph nodes, spleens and brain slices (Fig. 2a, b, c, d). The power of MB-SI-TPLSM is not only demonstrated by visualizing the fluorescence signal but also the SHG signal originating from collagen fibers (Fig. 2a, c). In addition to forming the extracellular matrix scaffold, collagen fibers are of central relevance due to their role as “highways” guiding cellular movement [36].

### Spatial resolution in tissue

**Figure 3:**
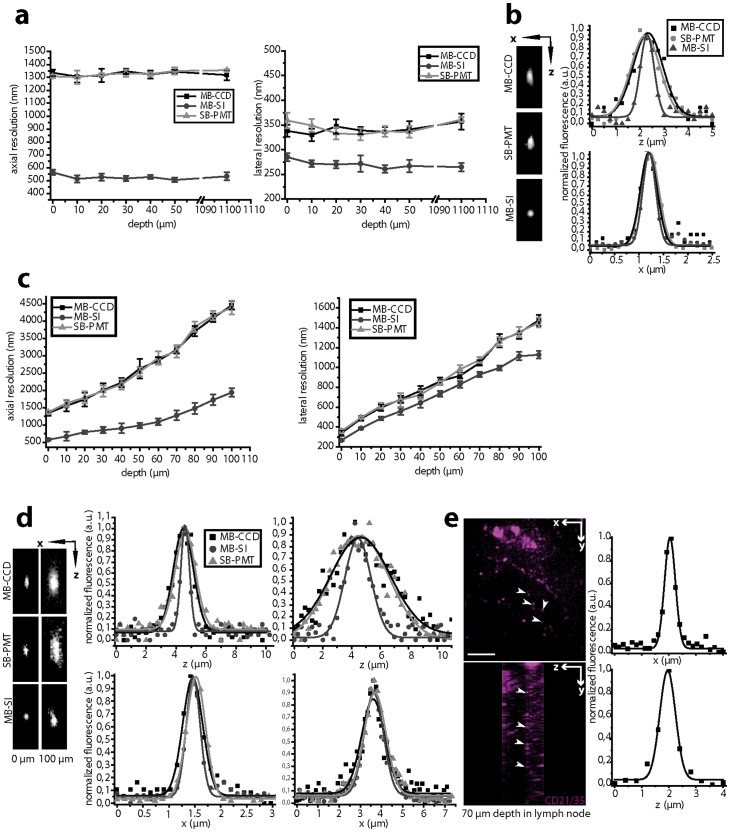
Depth-dependent spatial resolution in MB-SI-TPLSM. (**a**) Depth-dependence of both lateral and axial resolution of the fluorescence signal of 100 nm fluorescent beads (λ_em_ = 515 nm) recorded by SB-PMT-, MB-CCD- and MB-SI-TPLSM. λ_exc_ = 800 nm. (**b**) Representative lateral and axial profiles as well as xz-projections of the ePSFs acquired with the same set-ups. (**c**) Depth-dependence of both lateral and axial resolution of the fluorescence signal of 100 nm fluorescent beads (λ_em_ = 515 nm) in the same region of a lymph node recorded by SB-PMT-, MB-CCD- and MB-SI-TPLSM. λ_exc_ = 800 nm. (**d**) Representative lateral and axial profiles, as well as xz-projections of the ePSFs acquired by the same set-ups at the surface and in 100 µm depth in the lymph node. (**e**) xy-projection and corresponding yz-projection of a 3D image of anti-CD21/35-Fab-fragment-ATTO590 labeled follicular dendritic cells (FDCs) in a germinal center of the popliteal lymph node: intravital imaging by MB-SI-TPLSM in 80–95 µm depth. The arrowheads indicate clusters of labeled CD21/35. Their dimensions are depicted by typical profiles at the focal plane (x-profile) and along the optical axis of the microscope (z-profile). λ_exc_ = 860 nm, scale bar = 10 µm.

The spatial resolution of an imaging system is determined by the dimensions of the effective point spread function (ePSF) [14]. The ePSFs were measured by collecting either the local 3D-fluorescence signal of yellow-green fluorescent (505/515) 100 nm beads or the second harmonic generation (SHG) 3D-signal of collagen fibers in various samples using SB-PMT- TPLSM, MB-CCD-TPLSM and MB-SI-TPLSM (Fig. 3).

Although agarose media and collagen gels are appropriate for benchmarking experiments due to their easy handling (*Supplemental Material S1,* Fig. 3a,b, *Fig. S2*, Table 1), they do not fully reflect the situation in live tissue [14], [15]. We therefore compared the resolution performance of the three imaging setups in explanted lymph nodes and in popliteal lymph nodes of live mice.

**Table 1:** Lateral and axial resolutions measured with the 20× and 40× magnification objective lenses, respectively, on fluorescent micorbeads embedded in agarose or, for second harmonic generation, on collagen fibers in a collagen gel.

imaging depth /µm		20×, NA 0.95, WD = 2 mm						40×, NA 1.1, WD = 624 µm					
		fluorescence			SHG			fluorescence			SHG		
		PMT	CCD	SI	PMT	CCD	SI	PMT	CCD	SI	PMT	CCD	SI
	0	344	341	270	340	341	257	303	303	250	308	315	251
lateral		±13	±17	±17	±10	±9	±10	±6	±4	±9	±9	±28	±11
/nm	1100	361	357	265	331	337	262	309	312	265	-	-	-
	/400	±5	±16	±17	±5	±8	±9	±8	±7	±5			
	0	1331	1327	571	1339	1337	568	1031	1023	420	1033	1026	437
axial		±21	±45	±34	±44	±31	±29	±24	±24	±58	±20	±40	±29
/nm	1100	1337	1320	533	1346	1339	531	1037	1020	463	-	-	-
	/400	±34	±26	±66	±43	±43	±28	±34	±26	±23			

Measurements on highly-scattering lymph node tissue previously loaded with fluorescing 100 nm beads revealed a depth-dependent deterioration in axial and lateral resolution for all employed TPLSM setups (Fig. 3c,d). Hence, the spatial resolution at the surface is comparable to that measured in agarose. However, in 100 µm depth the lateral resolution deteriorated by a factor of 4.2 while the axial resolution became 3.3 times lower, independent of the imaging set-up. Due to a 2.4-fold improvement in axial resolution and 23% improved lateral resolution in MB-SI-TPLSM (Fig. 3c,d, Table 2) as compared to standard TPLSM, we are for the first time able to visualize clusters of antigen-carrying units (CD21/35 Fab-fragment Alexa568) at the contact region between follicular dendritic cells and germinal center B cells in 80–100 µm depth (Fig. 3e). Their dimensions as deducted from 3D reconstructions are 405±59 nm lateral and 545±117 nm axial (mean ± s.d.).

**Table 2:** Lateral and axial resolutions measured with the 20× and 40× magnification objective lenses, respectively, on fluorescent microbeads or, for second harmonic generation, on collagen fibers within lymph nodes.

	20×, NA 0.95, WD = 2 mm							
	imaging depth/µm	fluorescence			imaging depth/µm	SHG		
		PMT	CCD	SI		PMT	CCD	SI
lateral	0	359	340	267	5	365	384	251
/nm		±19	±29	±8		±22	±31	±23
	100	1451	1475	1128	45	744	738	560
		±49	±56	±39		±34	±28	±26
axial	0	1369	1341	572	5	1558	1536	636
/nm		±50	±75	±32		±84	±58	±25
	100	4386	4453	1836	85	3215	3255	1056
		±200	±123	±121		±130	±130	±70

	40×, NA 1.1, WD = 624 µm							
	imaging depth/µm	fluorescence			imaging depth/µm	SHG		
		PMT	CCD	SI		PMT	CCD	SI
lateral	10	317	336	267	0	308	303	254
/nm		±28	±20	±19		±11	±10	±8
	80	1034	1064	865	50	763	741	624
		±26	±55	±25		±27	±10	±18
axial	10	1402	1467	634	0	1046	1069	493
/nm		±54	±58	±62		±76	±102	±18
	80	3247	3120	1252	50	2436	2489	1111
		±62	±80	±121		±88	±89	±93

The resolution of the SHG signal of collagen fibers measured by the 20× objective lens and that of the fluorescence and SHG signal measured by the 40× objective lens in explanted lymph nodes confirmed our results of improved resolution by MB-SI-TPLSM (*Fig. S2*, Table 2). The pixel size varied also in this case between 90 and 160 nm. For each data point (each experimental setup and tissue depth with a tolerance of ±2.5 µm in *z*) 10–15 beads were analyzed.

### Depth-dependent SNR (ddSNR): image contrast and maximal penetration depth

**Figure 4:**
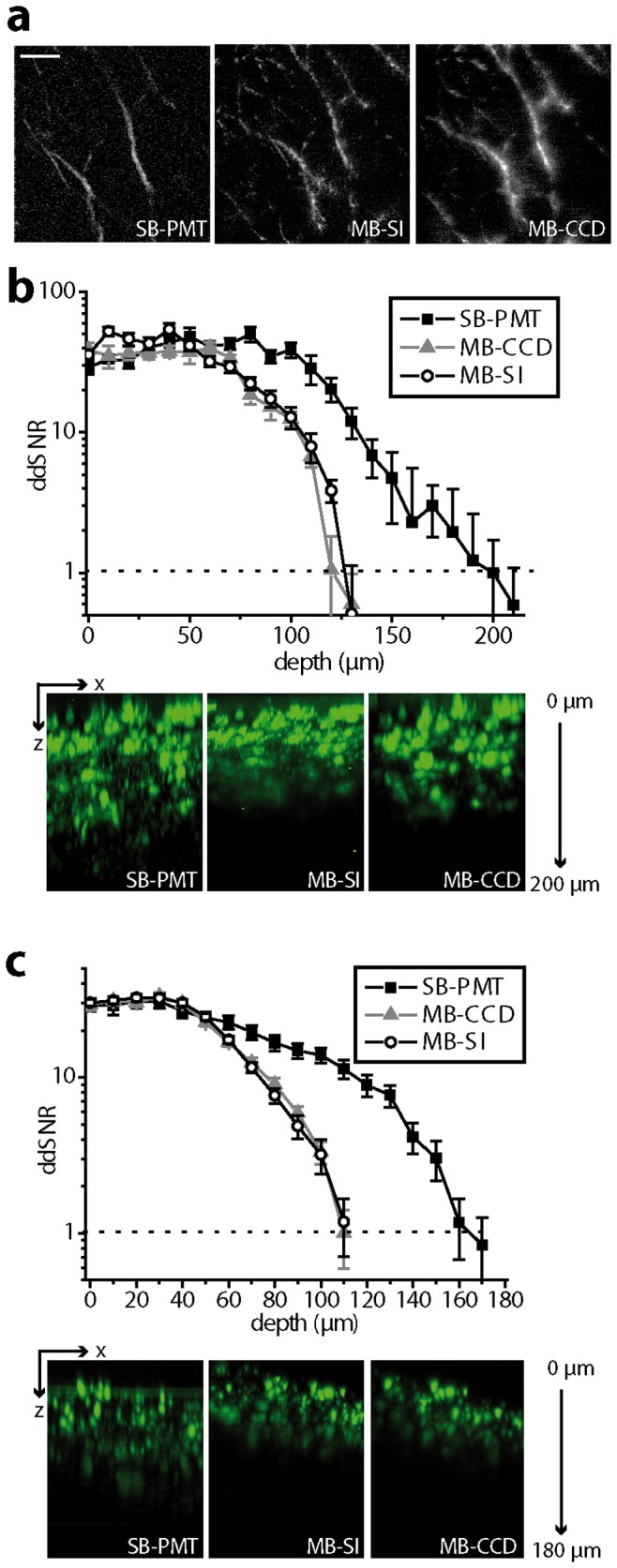
Depth-dependent SNR and maximal penetration depth in lymph node and spleen. (**a**) Second harmonic generation (SHG) images recorded in 80 µm depth at the same region of a lymph node with the MB-CCD-, SB-PMT- and MB-SI-TPLSM setups, respectively. The SHG signal mainly originates from fibrillar collagen. λ_exc_ = 900 nm, scale bar = 20 µm. (**b**) Depth-dependent signal-to-noise ratio recorded with the SB-PMT-, MB-CCD- and MB-SI-TPLSM at the same region of the spleen of an EGFP chimeric mouse and corresponding yz-projections of 3D fluorescence images in the spleen. (**c**) Depth-dependent signal-to-noise ratio recorded with the SB-PMT-, MB-CCD- and MB-SI-TPLSM at the same region in a lymph node of a B1–8^+/+^ Jk^−/−^ EGFP mouse and corresponding yz-projection of the 3D fluorescence images. λ_exc_ = 920 nm.

Besides spatial resolution, image quality in TPLSM is dependent on the image contrast which is quantified by the signal-to-noise ratio (SNR). We expect MB-SI-TPLSM to have an advantage over standard TPLSM in terms of contrast due to the differential principle of the MMA. The SNR values of the SHG signal of collagen fibers at the same region in 80 µm depth in lymph node (Fig. 4a) amount to 9.2±1.6 (MB-CCD-TPLSM), 5.3±1.9 (SB-PMT-TPLSM) and 12.4±0.9 (MB-SI-TPLSM) (mean ± s.d., 6 different areas). In all three cases, the experiments were performed at λ_exc_ = 900 nm with an excitation peak power of 5·10^5^ mW (corresponding to 5.93•10^25^ photons/µm^2^·s peak photon flux), an average power of 4.9 mW (corresponding to 5.91•10^17^ photons/µm^2^·s average photon flux) and similar acquisition times per frame (512×512 pixel). However, the contrast advantage of MB-SI-TPLSM over SB-PMT-TPLSM is limited by the decreasing SNR with imaging depth in tissue.

The maximum penetration depth is defined as the imaging depth in which the decreasing fluorescence/SHG signal reaches the level of the background noise, i.e. the depth-dependent signal to noise ratio (ddSNR) becomes 1. The maximal imaging depth in spleen tissue of EGFP chimeric mice is 35% larger when using PMT detection (200 µm) as compared to CCD detection (130 µm). The imaging depths using standard MB-CCD-TPLSM and MB-SI-TPLSM are similar, as shown in Fig. 4b and 4c. In lymph nodes of mice with EGFP expressing B cells, the imaging depth achieved with SB-PMT-TPLSM (160 µm) is 31% higher than that reached by MB-CCD or MB-SI-TPLSM (110 µm). Hence, the main limitation of MB-SI-TPLSM in deep-tissue imaging is related to the necessity for CCD detection. The employed mean laser power amounted to 2–3 mW in all depths. We avoided applying the well-established exponential increase of laser power with imaging depth in order to quantify the effect of refractive index mismatches in the tissue on the ddSNR without bias. Detailed information on the experimental parameters can be found in *Supplemental Material S1*.

### Dynamic intravital imaging in lymph node

Dynamic intravital two-photon imaging is used to investigate pathophysiologic mechanisms in real time and within a natural environment, i.e. the tissue of live animals. To speed up acquisition in order to analyze dynamics and communication of sub-cellular structures down to the level of single protein-complexes within the living organism is a great challenge.

**Figure 5:**
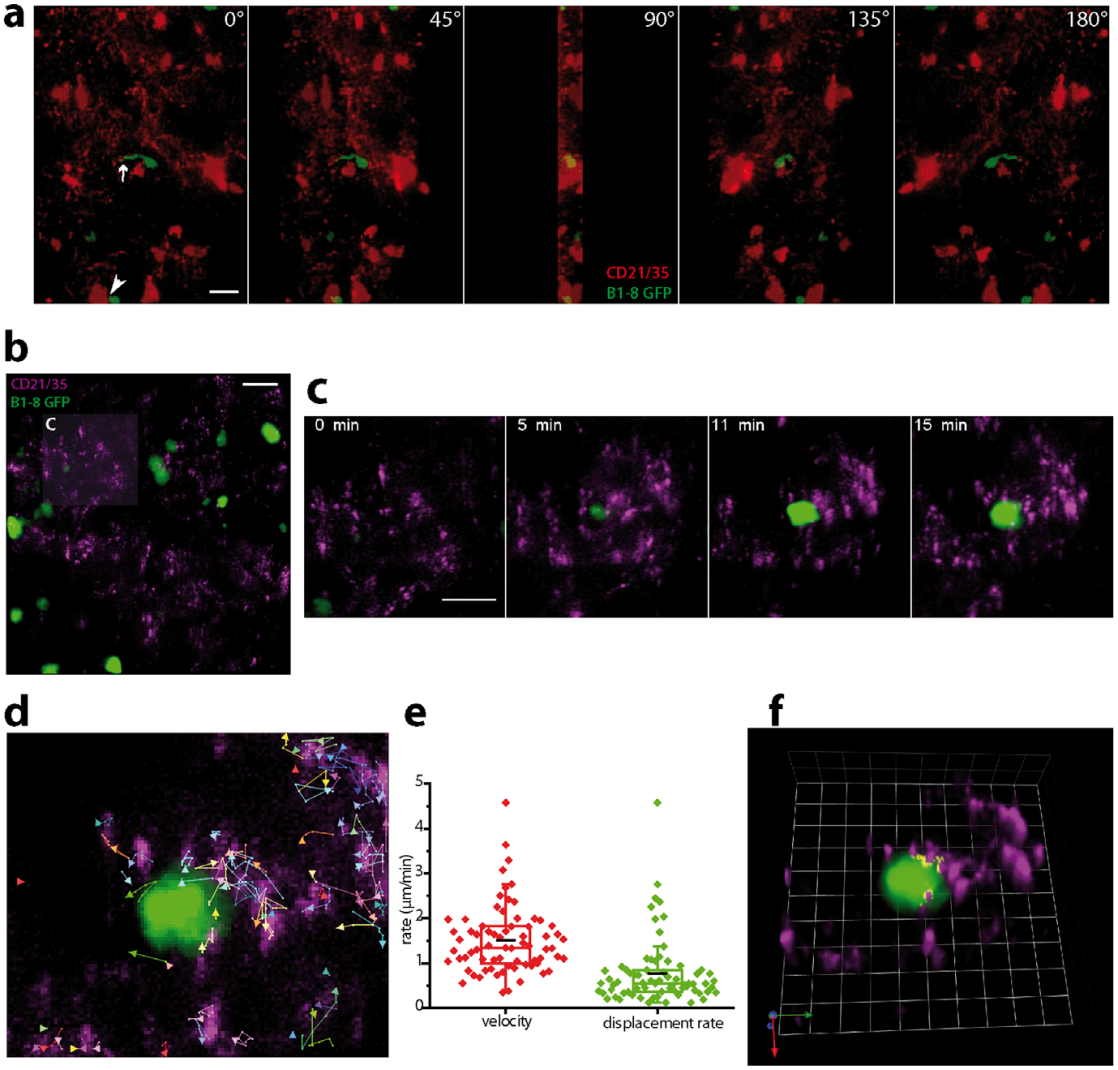
Dynamic intravital imaging by MB-SI-TPLSM. (**a**) Rotation view of a 3D fluorescence image in a germinal center of the popliteal lymph node of a mouse after footpad immunization with chicken γ-globulin (NP-CGG). Imaging was performed 9 days after immunization. Follicular dendritic cells (FDCs) were *in situ* stained by anti-CD21/CD35-Fab fragments coupled to Alexa568 (red) 24 h before imaging. NP-specific GFP^+^ (green) and GFP^−^ B cells were transferred into C57BL/6 mice. The communication between antigen-carrying FDC and B cells is thought to be involved in the clonal selection process of high affinity B cells. By means of MB-SI-TPLSM we were able to reveal the dynamic nature of the contacts between FDC somata and B cells (white arrowhead) but also, due to improved resolution and contrast, interactions between fine FDC processes and B cells (white arrow). λ_exc_ = 800 nm, scale bar = 10 µm. In the same context, we used for the staining of FDCs anti-CD21/CD35 Fab fragments coupled to ATTO590 for a better simultaneous visualization of FDCs and B cells (**b–d**). Time-lapse 3D fluorescence images by MB-SI-TPLSM in 85 to 105 µm depth in the lymph node (**c**) revealed that CD21/CD35+ immune complexes are accumulating around the B cells and that this interaction is highly dynamic (**d, e**). The trajectories of the CD21/35 clusters are shown in (**d**), whereas their statistics concerning velocity and displacement rate are summarized in (**e**). The contact regions between the CD21/35 clusters and B cell are shown in yellow (**f**). λ_exc_ = 860 nm, scale bar = 10 µm, grid unit = 3.5 µm.

Using MB-SI-TPLSM at the given spatial resolution, we are able to acquire z-stacks of 200×200×20 µm^3^ (500×500×21 voxel) in the popliteal lymph node of NP-CGG immunized live mice every 60 s over at least one hour (excitation peak power 5.26•10^5^ mW corresponding to a peak photon flux of 5.9•10^25^ photons/µm^2^·s at 850 nm or 8.03•10^5^ mW corresponding to a peak photon flux of 8.47•10^25^ photons/µm^2^·s at 800 nm). Thereby we are able to visualize and quantify the contacts between NP-specific B cells and FDCs within germinal centers at day 8 or 9 after immunization. The FDCs are stained *in vivo* with an anti-CD21/CD35-Fab-fragment conjugated to Alexa568 or to ATTO590 in order to visualize complement receptors, which are known to bind immune complexes on the FDC surface [2]. Using MB-SI-TPLSM we were able to visualize not only the interaction of B cells with FDC somata but also with their fine dendrites (*Movie S1* and *S2*). Even more, we are able to image the clusters of immune complexes on the FDCs via CD21/CD35 at the contact site between B cells and FDCs (Fig. 5a,b, *Fig. S3*).

In addition to B cells, which are known to actively move towards the FDCs, the FDC dendrites also show a high degree of motility (*Fig. S3*). Moreover, we are able to demonstrate for the first time that immune complexes clusters on the FDCs are also highly motile on the surface of the FDC membrane (Fig. 5c,d,e, *Movies S3, S4* and *S5*). We could exclude animal movement artifacts since the tracks of the CD21/35 signals are not unidirectional. The basis of this movement remains to be investigated.

### Photobleaching and photodamage

A main limitation in intravital time-lapse imaging is currently related to fluorescence signal loss due to fluorophore photobleaching, and the loss of functionality due to tissue photodamage. EGFP expressed by B cells within germinal centers of live mice did not show any photobleaching effects (*Fig. S4*) when MB-SI-TPLSM-based acquisition of 200×200×20 µm^3^ 3D images (500×500×21 voxel) was performed every 60 s over the course of 60 minutes. The excitation peak power at the sample surface was 6.2•10^5^ mW at 850 nm corresponding to a peak photon flux of 6.95•10^25^ photons/µm^2^·s, an average power of 6.08 mW and an average photon flux of 6.82•10^17^ photons/µm^2^·s. Moreover, the distribution of velocity and displacement rates of antigen-specific GFP+ cells within germinal centers measured by MB-SI-TPLSM (*Fig. S4*) is comparable to that previously measured by standard SB-PMT-TPLSM [4] confirming that cell migration is not affected. The time-lapse 3D-experiments were performed over 2 hours, every 60 s, at 850 nm excitation wavelength and 3.04•10^5^ mW peak power/3.69•10^25^ photons/µm^2^·s peak photon flux (2.98 mW average power/3.71•10^17^ photons/µm^2^·s average photon flux). Time-lapse experiments in *CerTN L15* mice led, even after 3 hours of illumination every 60 s, to no pathological increase of the neuronal calcium (over 1 µM) and, thus, to no dysfunction of the neurons. We conclude that MB-SI-TPLSM results in reduced photodamage, comparable to established PMT-based TPLSM techniques.

## Discussion

Although standard intravital imaging based on two-photon microscopy has answered many questions referring to cellular localization and motility patterns in the context of small animal pathophysiology [2]–[7], there are still limitations that prevent us from understanding and quantifying cellular communication. In particular, the depth-dependent deterioration of spatial resolution and loss of contrast (SNR) due to increased scattering in tissue [14], [15] make it difficult to unequivocally identify dynamic cellular interactions in living organisms.

Here, we present a novel approach termed striped-illumination two-photon microscopy, which is based on parallelized laser-scanning (MB-SI-TPLSM). The novel technique is able to improve the axial resolution up to 3-fold and to enhance contrast and lateral resolution (by up to 25%) deep within tissue as compared to standard TPLSM. Its power for intravital imaging is demonstrated by uncovering the dynamic nature of the communication between follicular dendritic cells (FDC) and B-cells within germinal centres (GCs) in the lymph nodes of living adult mice at the level of single immune complex clusters (few protein molecules). This communication is partially responsible for the clonal selection of B cells leading to affinity maturation of the immune response. In the future, this will be used to study the response of germinal center B cells following interactions with FDCs by probing cellular function, proliferation, differentiation or apoptosis.

While MB-SI-TPLSM clearly improves the image contrast in tissue, this advantage is limited by the SNR dependence on imaging depth, and by the extended acquisition time. As a field-detection-based technique, MB-SI-TPLSM cannot reach the imaging depth of SB-PMT-TPLSM, even if the excitation power is increased. Even if enough excitation photons (in NIR or IR range) reach deeper tissue areas, the stronger scattered emitted photons (in the visible range) are not reaching the detector in a proper way, thus a highly resolved correlation between emitted photon and its origin in the sample is no longer possible. Hence, in up to 120 µm of depth in lymph node tissue, the use of MB-SI-TPLSM leads to a significantly better image. In areas below 120 µm the contrast advantage of MB-SI-TPLSM is counteracted by the faster deterioration of SNR as compared to SB-PMT-TPLSM. This is due to scattering of only excitation light for the SB-PMT-TPLSM setup, whereas in the case of MB-SI-TPLSM both excitation and emission light scattering contribute to this effect. This rule generally holds true for other tissue types like spleen, brain, skin or bone marrow, while the critical imaging depth is strongly tissue-dependent. A parameter that characterizes this tissue-dependence is the mean scattering-free path, i.e. the exponential parameter of the laser power decay within a given tissue [37]. For instance, at 800 nm excitation wavelength the mean scattering-free path amounts to 20 µm [37] in lymph nodes, 47 µm in acute brain slices and to 200 µm in the brain cortex [38].

In terms of the acquisition rate, MB-SI-TPLSM benefits from parallelized excitation but looses speed as compared to standard MB-CCD-TPLSM since the approach is based on the acquisition of ∼10 serial images of the same area. However, it retains a clear speed advantage over SB-PMT-TPLSM.

As compared to single-photon-based excitation techniques (spinning disc confocal microscopy, versatile in achieving high image acquisition rates [39], or standard confocal microscopy), MB-SI-TPLSM retains both the benefits – e.g. deep-tissue imaging – and the short-comings – lower resolution – of near-infrared excitation characteristic for two-photon techniques. Due to the excitation at shorter wavelengths, both confocal and spinning disc confocal microscopy have a better diffraction limited resolution, i.e. at the surface of a specimen, in translucent samples [40] or in cell cultures, but are characterized by stronger scattering within tissue than any two-photon microscopy technique. The scattering of electromagnetic waves (of both excitation and emission light) within tissue leads to optical aberrations deteriorating both PSF (resolution) and SNR (contrast) with increasing imaging depths. The scattering on macromolecular structures scales with λ^2^, (λ being the excitation wavelength) [37]. Thus, we expect single-photon based techniques (excitation at λ = 532 nm) to achieve maximally a penetration depth of 53.5 µm within the lymph node as compared to a penetration depth of 160 µm attained by standard SB-PMT-TPLSM (excitation at 920 nm) at the same excitation intensity within the sample. This value is still half the penetration depth achieved by MB-SI-TPLSM (110 µm) confirming the superiority of our technique as a multi-focal TPLSM technique against established spinning disc single-photon excitation techniques [41].

In order to minimize the limiting effect of the visible emission wavelength in MB-SI-TPLSM on penetration depth, a promising option is to employ infra-red two-photon excitation of red or even near-infra-red fluorescing proteins and dyes. We previously showed that the depth-dependent deterioration of both spatial resolution and signal-to-noise ratio in tissue is significantly counteracted by the use of longer excitation wavelengths, i.e. infrared (IR) excitation of optical parametric oscillators (OPO), as compared to standard NIR (near infrared) excitation of Ti:Sa lasers [15]. This is due to the lower scattering in dense tissue at longer wavelengths and constitutes a great advantage both in terms of resolution and of imaging depth although, according to the diffraction theory, the resolution at these wavelengths is lower. In the future, the development of beam splitters which allow for parallelized scanning with IR radiation over a broad wavelength range will enable imaging at predicted resolutions of approx. 210 nm and 280 nm (lateral and axial, respectively) in 100 µm depth within lymph nodes by means of IR SI-TPLSM. The imaging depth is expected to be almost doubled (approximately 80% increase) [15].

In order to further improve lateral resolution (up to two-fold, theoretically), it is necessary to develop a Fourier-transform based evaluation approach containing correction algorithms for the movement within live animals.

We demonstrated that, during typical imaging sessions (60–180 illumination steps over the time of 1–3 hours), the chromophore photobleaching and tissue photodamage induced by MB-SI-TPLSM is negligible. The velocity and displacement rate of B cells in germinal centers and the neuronal Calcium level served as criteria to quantify photodamage. However, we cannot exclude the possibility that other more sensitive cellular parameters (e.g. the activation of repair enzymes) are affected.

The expected high impact of the striped-illumination approach for biosciences and biomedicine lies in its capacity to dramatically improve resolution of any kind of laser-scanning microscope, of which the confocal microscopes are the most prominent, by a simple technical adjustment, i.e. choosing an appropriate field detector.

## Supporting Information

Figure S1 **Lateral resolution of the fluorescence signal in multi-beam scanning TPLSM.** (**a**) Fluorescence images of 100 nm green fluorescing beads embedded in agarose gel, recorded by standard MB-CCD-TPLSM and by MB-SI-TPLSM. The evaluation was performed by the minimum-maximum algorithm (MMA) and by the Fourier-transform algorithm (FTA), respectively. λ_exc_ = 800 nm. (**b**) Corresponding profiles of two neighboring beads along the yellow line in (**a**).
(PDF)

Figure S2 **Spatial resolution by means of a 20× and a 40× water-immersion objective lens, respectively, in homogenous and heterogeneous media.** (**a**) Depth-dependence of lateral and axial resolution of the SHG signal of collagen fibers in gel (λ_SHG_ = 450 nm) recorded by SB-PMT-, MB-CCD- and MB-SI-TPLSM. (**b**) Corresponding comparative 3D SHG images in collagen gel. λ_exc_ = 900 nm. (**c**) Depth dependence of lateral and axial resolution of the SHG signal of collagen fibers in lymph node (λ_SHG_ = 450 nm) recorded by SB-PMT-, MB-CCD- and MB-SI-TPLSM. λ_exc_ = 900 nm. (**d**) Typical axial profiles of collagen fibers as recorded by SB-PMT-, MB-CCD- and MB-SI-TPLSM, and their corresponding xy and yz projections. (**e**) and (**f**) Depth-dependence of lateral and axial resolution of the fluorescence signal of 100 nm fluorescent beads in agarose medium and of SHG signal in collagen gel recorded at λ_exc_ = 800 nm and λ_exc_ = 900 nm using the 40× water-immersion objective lens (NA = 1.1). (**g**) and (**h**) Depth-dependence of lateral and axial resolution of the fluorescence signal of 100 nm fluorescent beads and of SHG signal of collagen in lymph nodes recorded at λ_exc_ = 800 nm and λ_exc_ = 900 nm using the same objective lens.
(PDF)

Figure S3 **Dynamic intravital imaging in germinal centers, in the popliteal lymph node by MB-SI-TPLSM.** (**a**) 3D imaging of follicular dendritic cells (FDCs) labeled with anti-CD21/35 Fab-fragment Alexa 568 and B1–8^+/+^ Jκ^−/−^ EGFP^+/+^ expressing cells in a germinal center of the popliteal lymph node of a mouse previously immunized with NP-CGG. (**b**) Time-lapse imaging revealing the dynamic interaction between the B cells and the soma of FDCs in germinal centers. (**c**) Time-lapse imaging revealing the interactions between B cells and the fine processes of FDCs. (**d**) Rotation views of a 3D fluorescence image in the germinal center of a mouse transferred with B1–8^+/+^ Jκ^−/−^ EGFP^+/+^ cells (green), *in situ* labeled with CD21/35 Fab-fragment Alexa 568 (FDCs in red) and immunized with NP-CGG.
(PDF)

Figure S4 **Photobleaching and photodamage in MB-SI-TPLSM.** (**a**) Tracking of B1–8^+/+^ Jκ^−/−^ EGFP^+/+^ cells within the germinal center light zone (FDC zone). 3D fluorescence image of FDCs (red) and B1–8^+/+^ Jκ^−/−^ EGFP^+/+^ cells (green) acquired by MB-SI-TPLSM, overlapped with the trajectories of the B cells recorded over 60 minutes (left). Distribution of the mean velocity and displacement rate of B cells within the germinal center (n = 30 cells). (**b**) Both the velocity and the displacement rate of the B cells in the germinal center 8 days after immunization with NP-CGG are comparable with values measured by standard SB-PMT-TPLSM. (**c**) Photobleaching of B1–8^+/+^ Jκ^−/−^ EGFP^+/+^ cells in the germinal center over 60 minutes. GFP expressed by B1–8^+/+^ Jκ^−/−^ EGFP^+/+^ cells shows only negligible photobleaching (**d**) during illumination of 200×200×20 µm^3^ 3D-stacks recorded each minute over the time course of an hour.
(PDF)

Movie S1 **Rotation view of a 3D fluorescence image (465×651×11 voxel, 186×260×10 µm^3^) in a germinal center of the popliteal lymph node of a NP-CGG immunized mouse, 9 days after immunization.** B1–8^+/+^ Jκ^−/−^ EGFP^+/+^ cells previously transferred to the C57BL6 mouse are represented in green and follicular dendritic cells (FDCs) *in situ* stained with anti-CD21/35-Fab fragment-Alexa568 are represented in red. λ_exc_ = 800 nm.
(MOV)

Movie S2 **Detail of movie S1.** Rotation view of a 3D fluorescence image (142×86×11 voxel, 57×34×10 µm^3^) of a germinal center in the popliteal lymph node of a NP-CGG immunized mouse, 9 days after immunization. B1–8^+/+^ Jκ^−/−^ EGFP^+/+^ cells previously transferred to the C57BL6 mouse are represented in green and follicular dendritic cells (FDCs) *in situ* stained with anti-CD21/35-Fab fragment-Alexa568 are represented in red. λ_exc_ = 800 nm.
(MOV)

Movie S3 **3D movie (480×480×7 voxel, 192×192×12 µm^3^) of fluorescence in a germinal center of the popliteal lymph node of a NP-CGG immunized mouse, 9 days after immunization.** B1–8^+/+^ Jκ^−/−^ EGFP^+/+^ cells previously transferred to the C57BL6 mouse are represented in green and follicular dendritic cells (FDCs) *in situ* stained with anti-CD21/35-Fab fragment-ATTO590 are represented in purple. λ_exc_ = 860 nm, time step = 60 s.
(MOV)

Movie S4 **(zoom-in of movie S3) Front view/3D movie (94×94×7 voxel, 38×38×12 µm^3^) of fluorescence in a germinal center of the popliteal lymph node of a NP-CGG immunized mouse, 9 days after immunization.** B1–8^+/+^ Jκ^−/−^ EGFP^+/+^ cells previously transferred to the C57BL6 mouse are represented in green and follicular dendritic cells (FDCs) *in situ* stained with anti-CD21/35-Fab fragment-ATTO590 are represented in purple. λ_exc_ = 860 nm, time step = 60 s.
(MOV)

Movie S5 **(zoom-in of movie S3) 3D movie (56×62×7 voxel, 22×25×12 µm^3^) of fluorescence in a germinal center of the popliteal lymph node of a NP-CGG immunized mouse, 9 days after immunization.** B1–8^+/+^ Jκ^−/−^ EGFP^+/+^ cells previously transferred to the C57BL6 mouse are represented in green and follicular dendritic cells (FDCs) *in situ* stained with anti-CD21/35-Fab fragment-ATTO590 are represented in purple. λ_exc_ = 860 nm, time step = 60 s, grid unit = 4.48 µm.
(MOV)

Supplemental Material S1 The section **Algorithms** describes the algorithms used to evaluate the set of striped-illumination fluorescence images in order to retrieve enhanced resolution and contrast: Min-Max Algorithm (MMA) and Fourier Transform Algorithm (FTA). In the section **Benchmarking** we compared in PSF experiments the performance of the MMA and FTA algorithms. In the additional **Material and Methods** we describe in more detail the animal surgery for imaging and the preparation of the explants used for the presented experiments. Detailed **Experimental Parameters** regarding the PSF, ddSNR and dynamic intravital imaging experiments are included. Further relevant literature is included in **Supplemental References**.
(DOCX)

## References

[1] Denk W, Strickler JH, Webb WW (1990) Two-photon laser scanning fluorescence microscopy. Science 248 4951: 73–6. 2321027 10.1126/science.2321027

[2] Hauser AE, Junt T, Mempel TR, Sneddon MW, Kleinstein SH, et al (2007) Definition of germinal-center B cell migration in vivo reveals predominant intrazonal circulation patterns. Immunity 26 5: 655–67. 17509908 10.1016/j.immuni.2007.04.008

[3] Cahalan MD, Parker I (2008) Choreography of cell motility and interaction dynamics imaged by two-photon microscopy in lymphoid organs. Annu Rev Immunol 26: 585–626. 18173372 10.1146/annurev.immunol.24.021605.090620 PMC2732400

[4] Germain RN, Bajenoff M, Castellino F, Chieppa M, Egen JG, et al (2008) Making friends in out-of-the-way places: how cells of the immune system get together and how they conduct their business as revealed by intravital imaging. Immunol Rev 221: 163–81. 18275481 10.1111/j.1600-065X.2008.00591.x

[5] Nimmerjahn A, Kirchhoff F, Helmchen F (2005) Resting microglial cells are highly dynamic surveillants of brain parenchyma in vivo. Science 308 5726: 1314–8. 15831717 10.1126/science.1110647

[6] Siffrin V, Radbruch H, Glumm R, Niesner R, Paterka M, et al (2010) In vivo imaging of partially reversible th17 cell-induced neuronal dysfunction in the course of encephalomyelitis. Immunity 33 3: 424–36. 20870176 10.1016/j.immuni.2010.08.018

[7] Esplugues E, Huber S, Gagliani N, Hauser AE, Town T, et al (2011) Control of Th17 cells occurs in the small intestine. Nature 475 7357: 514–8. 21765430 10.1038/nature10228 PMC3148838

[8] Helmchen F, Denk W (2005) Deep tissue two-photon microscopy. Nat Methods 2 12: 932–40. 16299478 10.1038/nmeth818

[9] Zipfel WR, Williams RM, Webb WW (2003) Nonlinear magic: multiphoton microscopy in the biosciences. Nat Biotechnol 21 11: 1369–77. 14595365 10.1038/nbt899

[10] Centonze VE, White JG (1998) Multiphoton excitation provides optical sections from deeper within scattering specimens than confocal imaging. Biophys J 75 4: 2015–24. 9746543 10.1016/S0006-3495(98)77643-X PMC1299873

[11] Rocheleau JV, Piston DW (2003) Two-photon excitation microscopy for the study of living cells and tissues. Curr Protoc Cell Biol Chapter 4: Unit 4 11. 10.1002/0471143030.cb0411s20 18228433

[12] Speier S, Nyqvist D, Cabrera O, Yu J, Molano RD, et al (2008) Noninvasive in vivo imaging of pancreatic islet cell biology. Nat Med 14 5: 574–8. 18327249 10.1038/nm1701 PMC3538807

[13] Niesner RA, Hauser AE (2011) Recent advances in dynamic intravital multi-photon microscopy. Cytometry A 79 10: 789–98. 21905212 10.1002/cyto.a.21140

[14] Niesner R, Andresen V, Neumann J, Spiecker H, Gunzer M (2007) The power of single and multibeam two-photon microscopy for high-resolution and high-speed deep tissue and intravital imaging. Biophys J 93 7: 2519–29. 17557785 10.1529/biophysj.106.102459 PMC1965440

[15] Herz J, Siffrin V, Hauser AE, Brandt AU, Leuenberger T, et al (2010) Expanding two-photon intravital microscopy to the infrared by means of optical parametric oscillator. Biophys J 98 4: 715–23. 20159168 10.1016/j.bpj.2009.10.035 PMC2820639

[16] de Gauw CJ, Vroom JM, van der Voorf HTM, Gerritsen HC (1999) Imaging properties in two-photon excitation microscopy and effects of refractive index mismatch in thick samples. Appl Opt 38 28: 5995–6003. 18324119 10.1364/ao.38.005995

[17] Fleire SJ, Goldman JP, Carrasco YR, Weber M, Bray D, et al (2006) B cell ligand discrimination through a spreading and contraction response. Science 312 5774: 738–41. 16675699 10.1126/science.1123940

[18] Hell SW, Rittweger E (2009) Microscopy: Light from the dark. Nature 461 7267: 1069–70. 19847257 10.1038/4611069a

[19] Eggeling C, Ringemann C, Medda R, Schwarzmann G, Sandhoff K, et al (2009) Direct observation of the nanoscale dynamics of membrane lipids in a living cell. Nature 457 7233: 1159–62. 19098897 10.1038/nature07596

[20] Westphal V, Rizzoli SO, Lauterbach MA, Kamin D, Jahn R, et al (2008) Video-rate far-field optical nanoscopy dissects synaptic vesicle movement. Science 320 5873: 246–9. 18292304 10.1126/science.1154228

[21] Gustafsson MG (2005) Nonlinear structured-illumination microscopy: wide-field fluorescence imaging with theoretically unlimited resolution. Proc Natl Acad Sci USA 102 37: 13081–6. 16141335 10.1073/pnas.0406877102 PMC1201569

[22] Gustafsson MG, Shao L, Carlton PM, Wang CJ, Golubovskaya IN, et al (2008) Three-dimensional resolution doubling in wide-field fluorescence microscopy by structured illumination. Biophys J 94 12: 4957–70. 18326650 10.1529/biophysj.107.120345 PMC2397368

[23] Mertz J (2011) Optical sectioning microscopy with planar or structured illumination. Nat Methods 8 10: 811–9. 21959136 10.1038/nmeth.1709

[24] Schermelleh L, Carlton PM, Haase S, Shao L, Winoto L, et al (2008) Subdiffraction multicolor imaging of the nuclear periphery with 3D structured illumination microscopy. Science 320 5881: 1332–6. 18535242 10.1126/science.1156947 PMC2916659

[25] Bates M, Huang B, Dempsey GT, Zhuang X (2007) Multicolor super-resolution imaging with photo-switchable fluorescent probes. Science 317 5845: 1749–53. 17702910 10.1126/science.1146598 PMC2633025

[26] Huang B, Wang W, Bates M, Zhuang X (2008) Three-dimensional super-resolution imaging by stochastic optical reconstruction microscopy. Science 319 5864: 810–3. 18174397 10.1126/science.1153529 PMC2633023

[27] Klein T, Loschberger A, Proppert S, Wolter S, van de Linde S, et al (2011) Live-cell dSTORM with SNAP-tag fusion proteins. Nat Methods 8 1: 7–9. 10.1038/nmeth0111-7b 21191367

[28] Moneron G, Hell SW (2009) Two-photon excitation STED microscopy. Opt Express 17 17: 14567–73. 19687936 10.1364/oe.17.014567

[29] Ding JB, Takasaki KT, Sabatini BL (2009) Supraresolution imaging in brain slices using stimulated-emission depletion two-photon laser scanning microscopy. Neuron 63 4: 429–37. 19709626 10.1016/j.neuron.2009.07.011 PMC2756148

[30] Bianchini P, Harke B, Galiani S, Vicidomini G, Diaspro A (2012) Single-wavelength two-photon excitation - stimulated emission depletion (SW2PE-STED) super-resolution imaging. Proc Natl Acad Sci USA 109 17: 6390–3. 22493221 10.1073/pnas.1119129109 PMC3340040

[31] Berning S, Willig KI, Steffens H, Dibaj P, Hell SW (2012) Nanoscopy in a living mouse brain. Science 335 6068: 551. 22301313 10.1126/science.1215369

[32] York AG, Parekh SH, Dalle Nogare DD, Fisher RS, Temprine K, et al (2012) Resolution doubling in live, multicellular organisms via multifocal structured illumination microscopy. Nat Methods 9: 749–54. 22581372 10.1038/nmeth.2025 PMC3462167

[33] Adie SG, Graf BW, Ahmad A, Carney PS, Boppart SA (2012) Computational adaptive optics for broadband optical interferometric tomography of biological tissue. Proc Natl Acad Sci USA 109 19: 7175–80. 22538815 10.1073/pnas.1121193109 PMC3358872

[34] Tang J, Germain RN, Cui M (2012) Superpenetration optical microscopy by iterative multiphoton adaptive compensation technique. Proc Natl Acad Sci USA 109 22: 8434–9. 22586078 10.1073/pnas.1119590109 PMC3365222

[35] Lu J, Min W, Conchello JA, Xie XS, Lichtman JW (2009) Super-resolution laser scanning microscopy through spatiotemporal modulation. Nano Lett 9 11: 3883–9. 19743870 10.1021/nl902087d PMC2783786

[36] Herz J, Paterka M, Niesner R, Brandt AU, Siffrin V, et al (2011) In vivo imaging of lymphocytes in the CNS reveals different behavior of naive T cells in health and autoimmunity. J Neuroinfl 8: 131. 10.1186/1742-2094-8-131 PMC3206448 21978405

[37] Caccia M, Sironi L, Collini M, Chirico G, Zanoni I, et al (2008) Image filtering for two-photon deep imaging of lymph nodes. Eur Biophys J 37 6: 979–87. 18389230 10.1007/s00249-008-0323-y

[38] Oheim M, Beaurepaire E, Chaigneau E, Mertz J, Charpak S (2001) Two-photon microscopy in brain tissue: parameters influencing the imaging depth. J Neurosci Methods 111 1: 29–37. 11574117 10.1016/s0165-0270(01)00438-1

[39] Wang E, Babbey CM, Dunn KW (2005) Performance comparison between the high-speed Yokogawa spinning disc confocal system and single-point scanning confocal systems. J Microscopy 218: 148–59. 10.1111/j.1365-2818.2005.01473.x 15857376

[40] Eisenhoffer GT, Rosenblatt J (2011) Live imaging of cell extrusion from the epidermis of developing zebra fish. J Vis Exp 2689. 21730948 10.3791/2689 PMC3197041

[41] Andresen V, Egner A, Hell SW (2001) Time-multiplexed multi-photon multi-focal microscopy. Opt Lett 26: 75–7. 18033511 10.1364/ol.26.000075

