# Supplementary figures and images for: High-Resolution Intravital Microscopy
Source: PLoS One. 2012 Dec 14;7(12):e50915. doi: 10.1371/journal.pone.0050915 (PMC3522675; doi:10.1371/journal.pone.0050915)

20x objective lens; NA = 0.95; WD = 2.0 mm; 100 nm fluorescent beads embedded in agarose

a

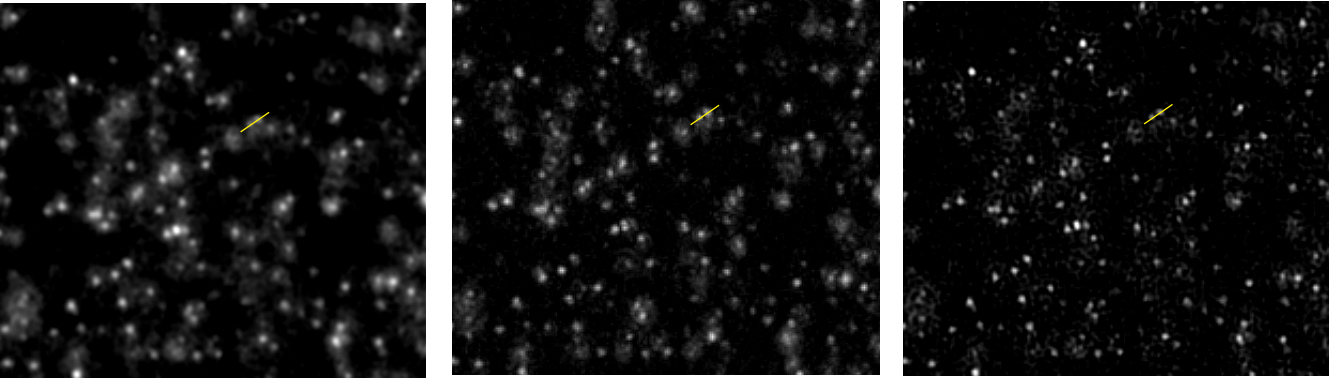

MB-CCD

MB-SI / MMA

MB-SI / FTA

b

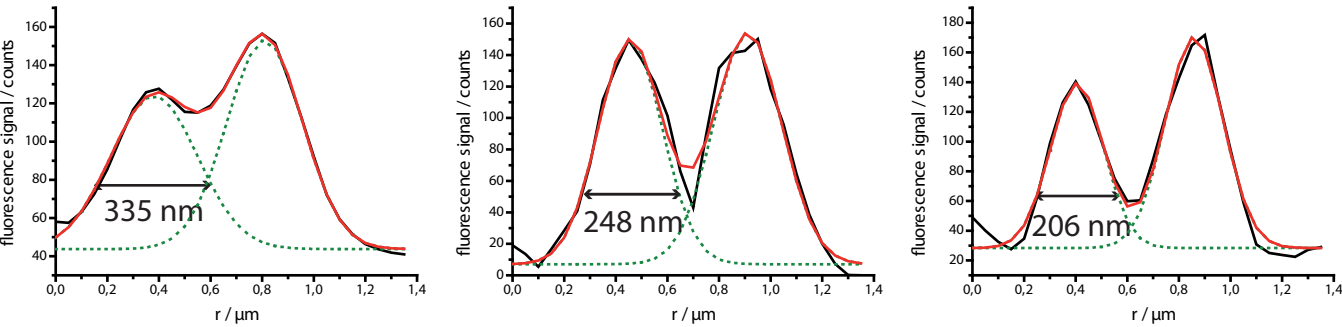

Supplement: Figure S1 — Lateral resolution of the fluorescence signal in multi-beam scanning TPLSM. (a) Fluorescence images of 100 nm green fluorescing beads embedded in agarose gel, recorded by standard MB-CCD-TPLSM and by MB-SI-TPLSM. The evaluation was performed by the minimum-maximum algorithm (MMA) and by the Fourier-transform algorithm (FTA), respectively. λexc = 800 nm. (b) Corresponding profiles of two neighboring beads along the yellow line in (a). (PDF) [file pone.0050915.s001.pdf]

20x objective lens; NA = 0.95; WD = 2.0 mm

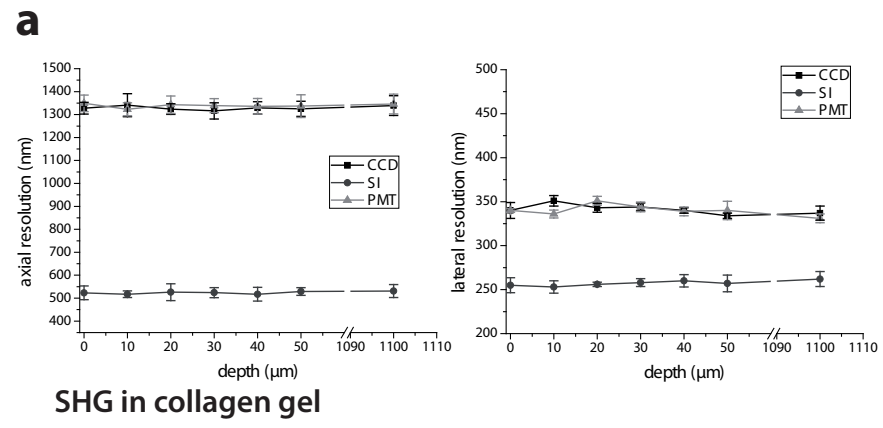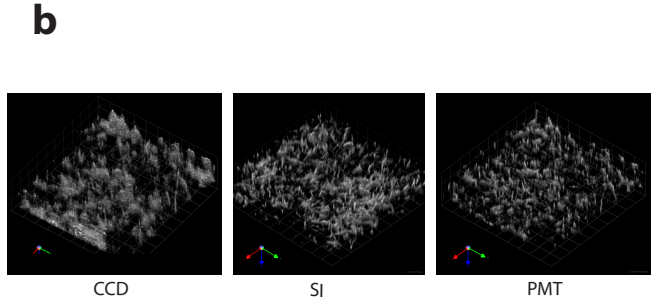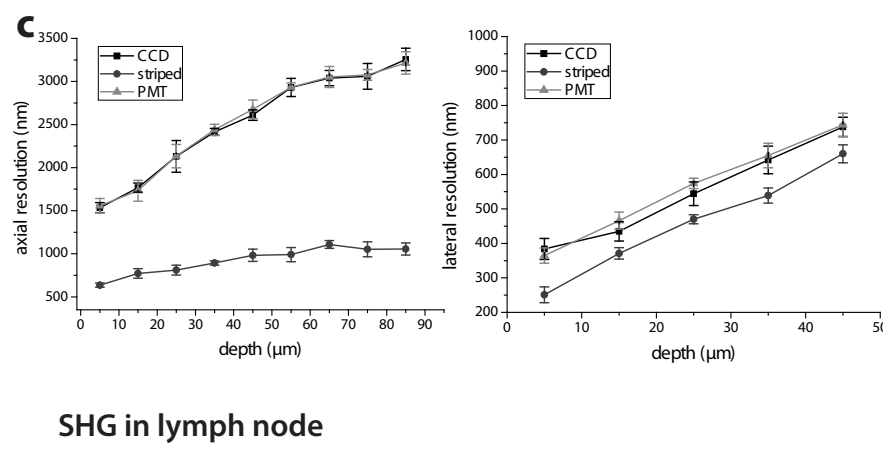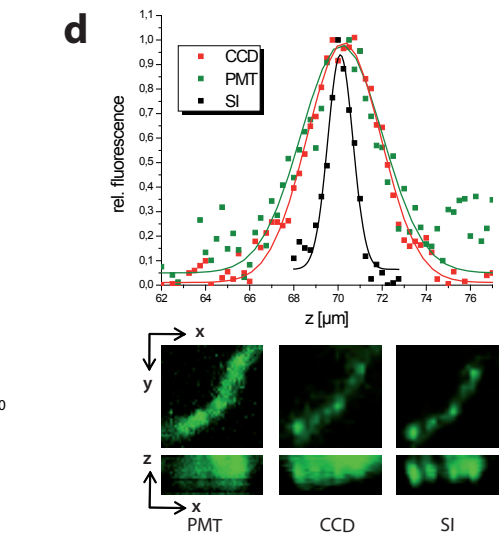

40x objective lens; NA = 1.1; WD = 0.624 mm

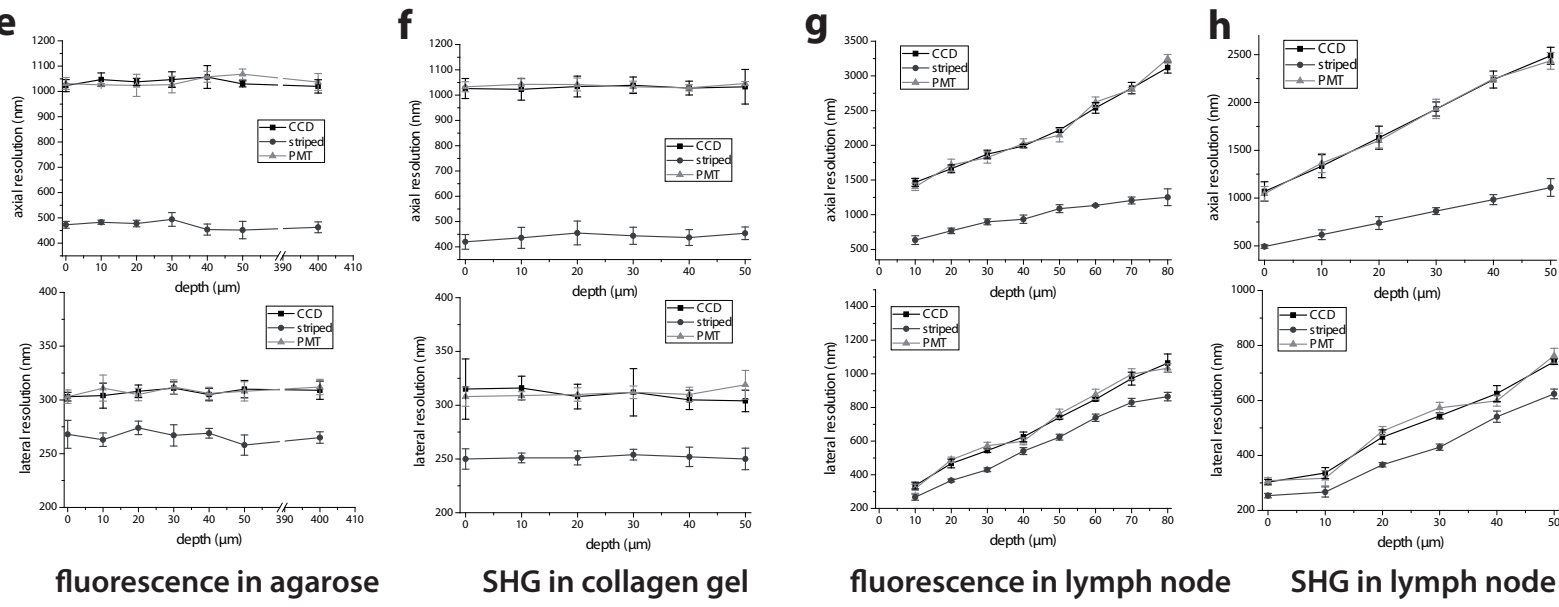

Supplement: Figure S2 — Spatial resolution by means of a 20× and a 40× water-immersion objective lens, respectively, in homogenous and heterogeneous media. (a) Depth-dependence of lateral and axial resolution of the SHG signal of collagen fibers in gel (λSHG = 450 nm) recorded by SB-PMT-, MB-CCD- and MB-SI-TPLSM. (b) Corresponding comparative 3D SHG images in collagen gel. λexc = 900 nm. (c) Depth dependence of lateral and axial resolution of the SHG signal of collagen fibers in lymph node (λSHG = 450 nm) recorded by SB-PMT-, MB-CCD- and MB-SI-TPLSM. λexc = 900 nm. (d) Typical axial profiles of collagen fibers as recorded by SB-PMT-, MB-CCD- and MB-SI-TPLSM, and their corresponding xy and yz projections. (e) and (f) Depth-dependence of lateral and axial resolution of the fluorescence signal of 100 nm fluorescent beads in agarose medium and of SHG signal in collagen gel recorded at λexc = 800 nm and λexc = 900 nm using the 40× water-immersion objective lens (NA = 1.1). (g) and (h) Depth-dependence of lateral and axial resolution of the fluorescence signal of 100 nm fluorescent beads and of SHG signal of collagen in lymph nodes recorded at λexc = 800 nm and λexc = 900 nm using the same objective lens. (PDF) [file pone.0050915.s002.pdf]

Time-lapse SI-MB-TPLSM imaging in germinal centers in the popliteal lymph node

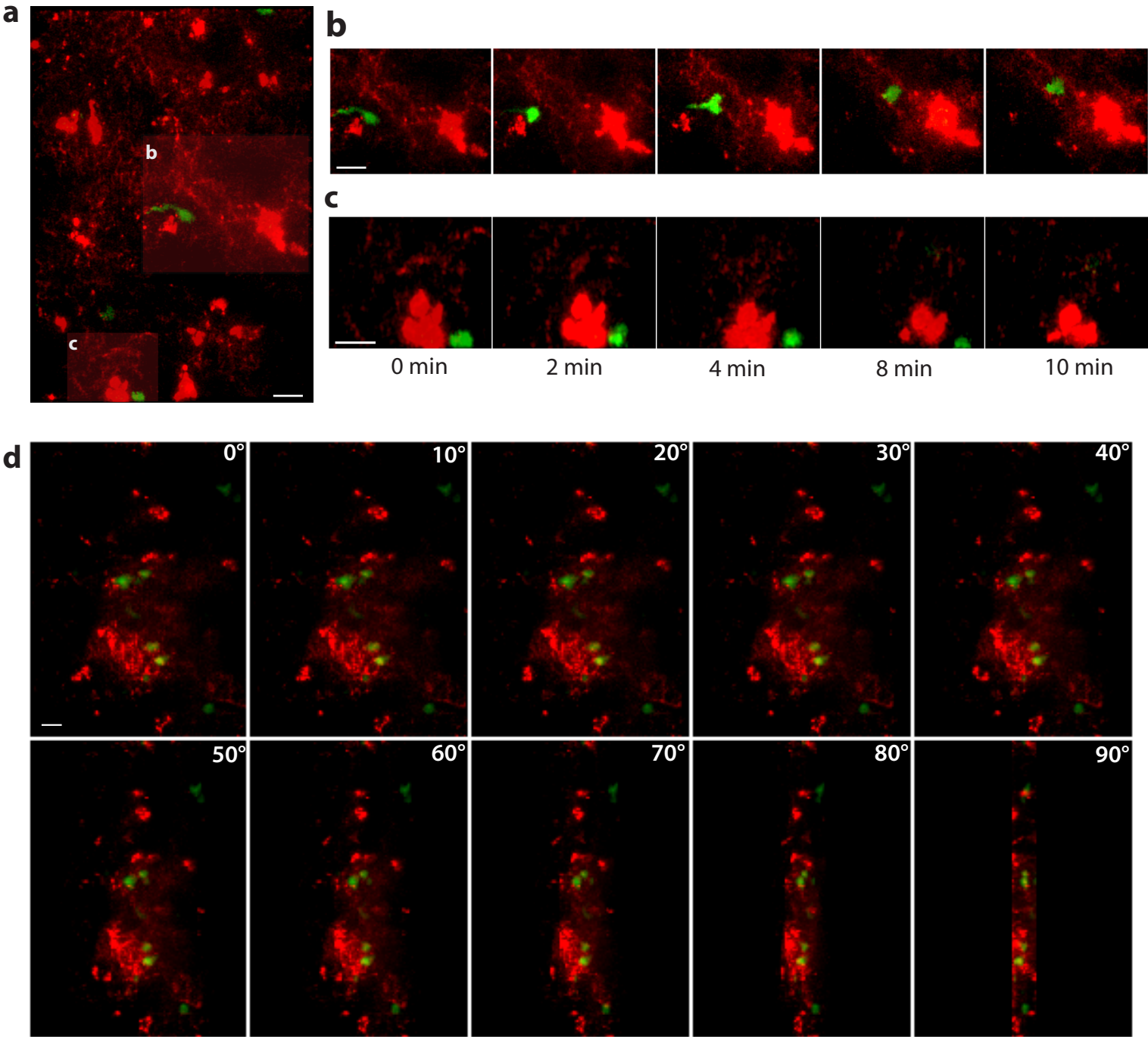

Supplement: Figure S3 — Dynamic intravital imaging in germinal centers, in the popliteal lymph node by MB-SI-TPLSM. (a) 3D imaging of follicular dendritic cells (FDCs) labeled with anti-CD21/35 Fab-fragment Alexa 568 and B1–8+/+ Jκ−/− EGFP+/+ expressing cells in a germinal center of the popliteal lymph node of a mouse previously immunized with NP-CGG. (b) Time-lapse imaging revealing the dynamic interaction between the B cells and the soma of FDCs in germinal centers. (c) Time-lapse imaging revealing the interactions between B cells and the fine processes of FDCs. (d) Rotation views of a 3D fluorescence image in the germinal center of a mouse transferred with B1–8+/+ Jκ−/− EGFP+/+ cells (green), in situ labeled with CD21/35 Fab-fragment Alexa 568 (FDCs in red) and immunized with NP-CGG. (PDF) [file pone.0050915.s003.pdf]
