# Supplementary material for: High-Resolution Intravital Microscopy
Source: PLoS One. 2012 Dec 14;7(12):e50915. doi: 10.1371/journal.pone.0050915 (PMC3522675; doi:10.1371/journal.pone.0050915)

B1-8 GFP+ cells dynamics in the germinal center

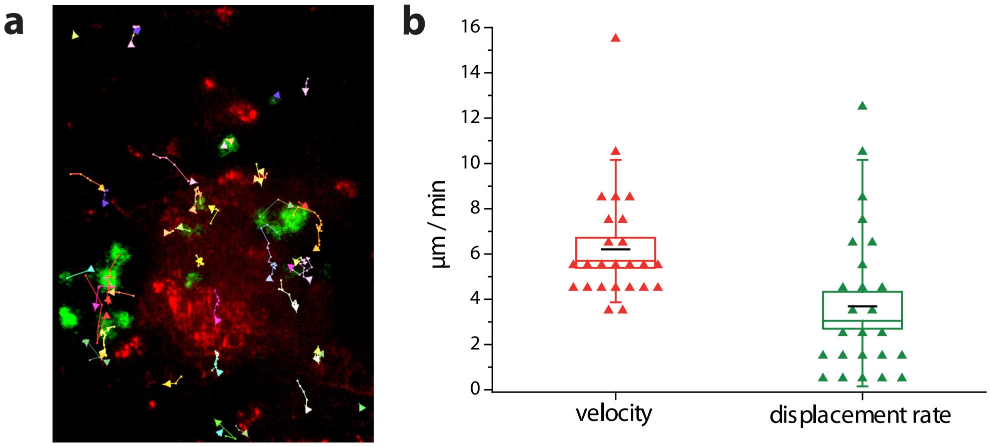

Photobleaching of GFP expressed in B1-8 cells by SI-MB-TPLSM

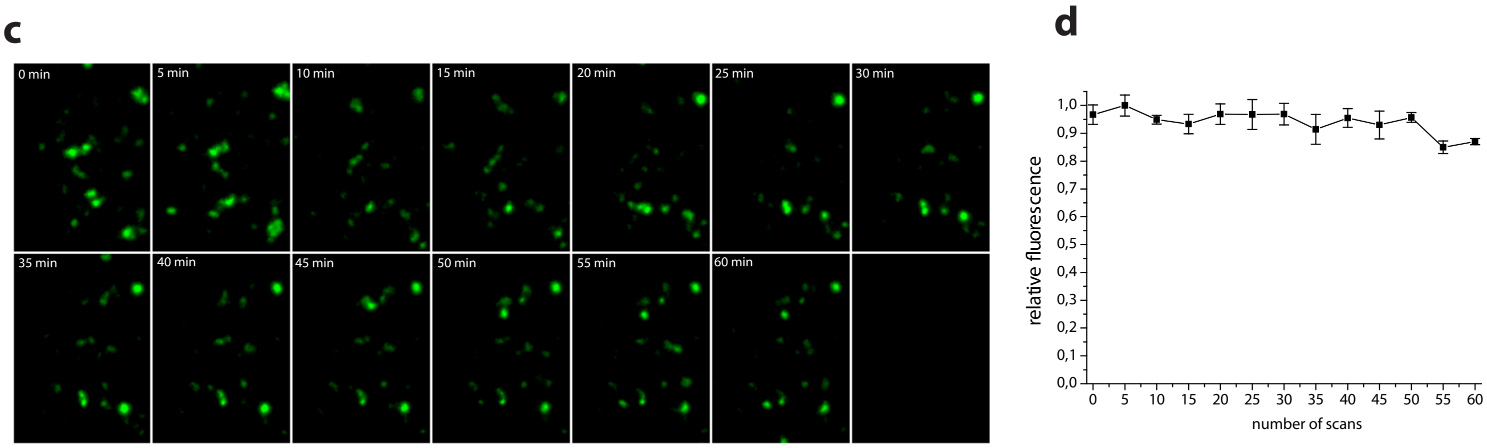

Supplement: Figure S4 — Photobleaching and photodamage in MB-SI-TPLSM. (a) Tracking of B1–8+/+ Jκ−/− EGFP+/+ cells within the germinal center light zone (FDC zone). 3D fluorescence image of FDCs (red) and B1–8+/+ Jκ−/− EGFP+/+ cells (green) acquired by MB-SI-TPLSM, overlapped with the trajectories of the B cells recorded over 60 minutes (left). Distribution of the mean velocity and displacement rate of B cells within the germinal center (n = 30 cells). (b) Both the velocity and the displacement rate of the B cells in the germinal center 8 days after immunization with NP-CGG are comparable with values measured by standard SB-PMT-TPLSM. (c) Photobleaching of B1–8+/+ Jκ−/− EGFP+/+ cells in the germinal center over 60 minutes. GFP expressed by B1–8+/+ Jκ−/− EGFP+/+ cells shows only negligible photobleaching (d) during illumination of 200×200×20 µm3 3D-stacks recorded each minute over the time course of an hour. (PDF) [file pone.0050915.s004.pdf]
