# Supplementary material for: High-Resolution Intravital Microscopy
Source: PLoS One. 2012 Dec 14;7(12):e50915. doi: 10.1371/journal.pone.0050915 (PMC3522675; doi:10.1371/journal.pone.0050915)
Supplement: Supplemental Material S1 — The section Algorithms describes the algorithms used to evaluate the set of striped-illumination fluorescence images in order to retrieve enhanced resolution and contrast: Min-Max Algorithm (MMA) and Fourier Transform Algorithm (FTA). In the section Benchmarking we compared in PSF experiments the performance of the MMA and FTA algorithms. In the additional Material and Methods we describe in more detail the animal surgery for imaging and the preparation of the explants used for the presented experiments. Detailed Experimental Parameters regarding the PSF, ddSNR and dynamic intravital imaging experiments are included. Further relevant literature is included in Supplemental References. (DOCX) [file pone.0050915.s010.docx]

**Supplemental Material**

**High-resolution intravital microscopy**

Volker Andresen2§, Karolin Pollok1,3§, Jan-Leo Rinnenthal3,1, Laura Oehme1, Robert Günther1, Heinrich Spiecker2, Helena Radbruch3,1, Jenny Gerhard1,3, Anje Sporbert4, Zoltan Cseresnyes4, Anja E. Hauser1* and Raluca Niesner1,3*

*1 German Rheumatism Research Center, Berlin Germany*

*2 LaVision Biotec GmbH, Bielefeld Germany*

*3Charité – University of Medicine, Berlin Germany*

*4 Max-Delbrück Center for Molecular Medicine, Berlin Germany*

*§* equally contributing § first and *senior authors*

**Address correspondence to:**

Dr. Raluca Niesner, German Rheumatism Research Center, Charitéplatz 1, 10117 Berlin, Germany

Phone: +49-30-28460-683; Fax: +49-30-+49-30-28460-604; E-mail: [niesner@drfz.de](mailto:niesner@drfz.de)

**Summary:**

**Algorithms**

**Benchmarking**

**Material and Methods**

**Experimental Parameters**

**Supplemental References**

**ALGORITHMS**

**Evaluation algorithms for MB-SI-TPLSM**

The image *I(r,z)* of an unknown structure *s(r,z)* as acquired by standard TPLSM is given by: *I(r,z)* = *s(r,z)*⊗*PSF(r,z)* with *PSF(r,z)* the effective point spread function, with *r² = x² + y²*. As observed in experiments on sub-resolution fluorescent microbeads, we can simplify the Bessel-function (*J_1_*)-based analytic formula and approximate the PSF by a 3D-Gaussian function:

$$PSF\left( r,z \right)=e^{-r^{2}/w^{2}}\cdot e^{-z^{2}/zw^{2}} .$$

Thus, the optical transfer function (Fourier-transform of PSF) is also a 3D-Gaussian function:

$$OTF\left( k_{r},k_{z} \right)={\sqrt{\pi\cdot w^{2}}\cdot\sqrt{\pi\cdot zw^{2}}\cdot e}^{-{\pi^{2}\cdot k}_{r}^{2}\cdot w^{2}}\cdot e^{-{\pi^{2}\cdot k}_{z}^{2}\cdot zw^{2}} .$$

The images generated by illuminating the structure in the sample with the striped-illumination pattern are given by: *SI*_t_*(x+t·h,y,z)* = [*p(x+t·h,y,z)· s(x,y,z)*]⊗*PSF(x,y,z)* with *t* between 1 and *n* number of translations and *h* the step between two consecutive positions of the pattern. We have shown that in agarose gel we can assume a known *PSF(x,y,z)* (3), which premise is used in the following calculation. However, in tissue the PSF dramatically changes within an image and even more with increasing penetration depth.

Different to structured illumination microscopy, the normalized pattern term *p_t_(x,y,z)* = *p(x+t·h,y,z)* is:

$$p_{t}\left( x,y,z \right)=\left\{ \begin{aligned} C\cdot\delta(z)\cdot\sum_{j=0}^{m} \delta(x+j\cdot r), &y<y_{start\_scan} \\ 0, &y>y_{end\_scan} \end{aligned} \right.$$

with *r* the known distance between the beam lets within the line (2.8 µm / 112 steps of the scanner for the 20x objective lens), *C* a constant given by the experimental setup and by the molecular properties, *δ* the unitary Dirac delta function, *h* the translation step of 175 nm and *t* varying between 0 and 15.

Minimum-maximum algorithm (MMA)

A robust algorithm to achieve resolution improvement (applies only for axial resolution) is to compare in each (x,y) pixel of the image the values of *SI*_t_*(x,y,z)* and to determine the maximum and the minimum value. In the resulting image, the attributed value is *max(SI*_t_*(x,y,z)) - min(SI*_t_*(x,y,z))*.

Fourier transform algorithm (FTA)

In order to explicitly improve the lateral resolution, the series of images *SI*_t_*(r,z)* is Fourier-transformed by an FFT algorithm in ${SI}_{t}\left( k_{r},k_{z} \right)$.

The ${SI}_{t}\left( k_{r},k_{z} \right)$ images are phase-shifted corresponding to *h* and combined to the final image using a generalized Wiener-filter, similar to (1):

$$s\left( k_{r},k_{z} \right)=\frac{\sum_{t} {OTF\left( k_{r},k_{z} \right)\cdot SI}_{t}\left( k_{r},k_{z} \right)}{\sum_{t} OTF\left( k_{r},k_{z} \right)+\omega^{2}}\cdot A(k_{r},k_{z})$$

with A($k_{r},k_{z})$ an apodization function (linear triangular function) and $\omega$ the Wiener factor which was empirically determined to achieve best lateral resolution. $s\left( k_{r},k_{z} \right)$ was then transformed back to the Cartesian space.

To improve the algorithm for maximum resolution enhancement, analytic computation of the optimal shifting parameters using the multi-Gaussian excitation pattern $p\left( x+t\cdot h,y,z \right)$ instead of the sum of harmonics as in structured-illumination is necessary. This is why the theoretically expected 2-fold improvement of lateral resolution as compared to standard TPLSM is not achieved: for an excitation wavelength of 800 nm the expected lateral resolution in TPLSM is 370 nm, using SI-TPLSM in combination with FTA evaluation a resolution of 185 nm is predicted.

Alternatively, the excitation pattern can be additionally rotated in two (π/2) or three directions (2π/3) to avoid artifacts due to the improvement of lateral resolution in only one direction after evaluation with the Fourier-transform algorithm (1).

**BENCHMARKING**

**Supplemental SI-TPLSM: spatial resolution and evaluation algorithms**

The lateral resolution amounts to 331 ± 20 nm if measured by MB-CCD-TPLSM, to 203 ± 17 nm if measured by MB-SI-TPLSM and evaluated by FTA (40% improvement) and to 277 ± 23 nm if measured by MB-SI-TPLSM and evaluated by MMA (16% improvement). Using the FTA evaluation we expect an improvement of 50% for the lateral resolution. λ_exc_ = 800 nm; 100 nm green fluorescing beads.

**PSF benchmarking in artificial media: agarose gel and collagen gel**

The ePSFs were measured by collecting either the local 3D-fluorescence signal of yellow-green fluorescent (505/515) 100 nm beads in agarose gel or the 3D second harmonic generation (SHG) signal of fibers in collagen gel matrix using SB-PMT-TPLSM, MB-CCD-TPLSM and MB-SI-TPLSM.

We measured the same lateral and axial resolution using PMT detection and standard CCD detection but up to 28% better lateral resolution and 2.7 fold better axial resolution when using MB-SI-TPLSM at λ_exc_ = 800 nm in agarose gel (Fig. 3b, *Table 1*). The prediction of the paraxial approximation (370 nm lateral and 1329 nm axial resolution) agrees well with the values measured using standard SB-PMT- and MB-CCD-TPLSM. Independent of the set-up, spatial resolution did not change with increasing penetration depth down to 1,100 µm (Fig. 3a).

In collagen gels, the resolution of the SHG signal shows a similar behavior. An improvement of 25% in lateral direction and 2.4-fold axially by MB-SI-TPLSM as compared to standard TPLSM techniques was achieved at λ_exc_ = 900 nm (*Suppl. Fig. 2*, *Table 1*).

A clear benefit in resolution compared to the 20x objective lens (NA = 0.95, working distance = 2 mm) typically used in intravital microscopy can be achieved by a 40x water-immersion objective lens (NA = 1.1, working distance = 624 µm). Similar results were achieved also under these conditions for both fluorescence and SHG signal, i.e. 17% - 20% better lateral resolution and 2.2 to 2.45 fold better axial resolution by SI-MB-TPLSM, respectively (*Suppl. Fig. 2* and *Table 1*). The pixel size varied between 90 and 160 nm.

**MATERIAL and METHODS**

**Mice**

Chimeric mice expressing EGFP under the Ubiquitin promoter were irradiated in order to delete the hematopoietic compartment. The mice were reconstituted with a non-fluorescent immune system. B1-8^+/+^ Jκ^-/-^ mice express EGFP under the Ubiquitin promoter. CerTN L15 mice (kindly provided by Oliver Griesbeck) express a FRET-based Ca^2+^-biosensor under the Thy1-expression cassette. The Ca^2+^-biosensor is based on Cerulean (CFP derivative) and Citrine (YFP derivative), as a FRET pair, bound to Troponin C, a muscle protein with four Ca^2+^ binding sites (2).

**Brain slice culture**

Acute hippocampus slices were harvested and prepared from adult mice (6 – 8 weeks). Subsequently they were imaged as previously described (3,4).

**Lymph node preparation**

For the determination of resolution in explanted lymph nodes, 10 µl of a 2% suspension of 100 nm fluorescent beads in phosphate buffer solution (PBS) (emission at 515 nm, Invitrogen, Germany) are injected in the footpad of C57Bl/6 mice. After 2 hours, mice were sacrificed and lymph nodes isolated, placed on a glass slide in anti-fading medium (DMEM^gfp^, Evrogen) and imaged.

**Spleen preparation**

Chimeric mice expressing eGFP under the Ubiquitin promoter in all non-hematopoietic cells and reconstituted with non-fluorescent hematopoietic cells were sacrificed and their spleen was removed and immediately imaged.

**Immune response in the popliteal lymph node: induction and preparation for intravital imaging**

The induction of a germinal center response in the popliteal lymph node and preparation of the imaging field was performed according to Hauser et al. (5). In brief, B cells were immunomagnetically purified from spleens of B1-8^+/+^ Jκ^-/-^ and B cells of B1-8^+/+^ Jκ^-/-^ GFP^+^ mice using the EasySep Negative Selection Mouse B Cell Enrichment Kit (StemCell Technologies). 3·10^6^ NP-specific B cells with a purity of >95% were injected intravenously using 1/8 B1-8^+/+^ Jκ^-/-^ GFP^+^ and 7/8 nonfluorescent B1-8^+/+^ Jκ^-/-^ in C57Bl/6 recipients. One day after cell transfer the recipients were immunized with 10 µg of NP-CGG (nitrophenyl-chicken ɣ globulin) emulsified in Complete Freund´s adjuvant (CFA) in the right hind food pad.

Fab fragments of anti-CD21/CD35 antibodies were labelled with Alexa-dye AL568 (Invitrogen, Germany) or ATTO590 (ATTOTech, Tübingen, Germany) succinimidyl esters. To highlight the network of follicular dendritic cells (FDC) within the germinal center *in vivo*, 10 µg of fluorochrome-labeled Fab fragments were injected into the right hind footpad of the mice 12 - 24 h before intravital analysis was performed.

Intravital investigations were performed 8 or 9 days after immunization. Mice were anaesthetized by i.p. injection of ketamin/xylazin, the amount depending on their weight. Reflexes were tested to monitor the depth of anesthesia over the whole imaging period. The anaesthetized animal was transferred to a custom-built surgery and microscopy platform and fixed. The popliteal lymph node was exposed, kept moist using 0.9% NaCl and covered with a glass cover slip. A temperature of 37°C was maintained at all times during imaging using a heating coil, also the body temperature was maintained at 37°C. After each imaging experiment, mice were sacrificed.

**EXPERIMENTAL PARAMETERS**

**Depth-dependent SNR (ddSNR)**

The excitation of EGFP was performed at 3.11∙10^5^ mW peak power / 3.71∙10^25^ photons/µm²·s peak photon flux in the SB-PMT-TPLSM measurements (3 mW average power / 3.64∙10^17^ photons/µm²·s average photon flux) and at 3.04∙10^5^ mW peak power / 3.69∙10^25^ photons/µm²·s peak photon flux (2.98 mW average power / 3.71∙10^17^ photons/µm²·s average photon flux) in the MB-CCD-TPLSM and MB-SI-TPLSM measurements on spleen tissue at 920 nm wavelength. In the experiments on lymph nodes, the applied peak power was 2.13∙10^5^ mW (2.49∙10^25^ photons/µm²·s peak photon flux) in single-beam mode (SB-PMT-TPLSM; average power 2.01 mW; average photon flux 2.44∙10^17^ photons/µm²·s) and 2.19∙10^5^ mW (2.66∙10^25^ photons/µm²·s peak photon flux) in multi-beam mode (MB-CCD-TPLSM and MB-SI-TPLSM; average power 2.15 mW; average photon flux 2.61∙10^17^ photons/µm²·s) also at 920 nm. The excitation power was measured directly at the specimen surface in all cases. The laser dwell times per pixel were chosen in a way that similar SNR values were achieved at the surface for all three setups. Although an exponential increase of the mean excitation power with increasing imaging depth in tissue (6) is widely used, we omitted this step to avoid artifacts caused by a more complicated evaluation of the ddSNR.

**REFERENCES**

1. Gustafsson MG, Shao L, Carlton PM, Wang CJ, et al. (2008) Three-dimensional resolution doubling in wide-field fluorescence microscopy by structured illumination. Biophys J. 94(12): 4957-70.
2. Heim N, Garaschuk O, Friedrich MW, Mank M, et al. (2007) Improved calcium imaging in transgenic mice expressing a troponin C-based biosensor. Nat Methods. 4(2): 127-9.
3. Herz J, Siffrin V, Hauser AE, Brandt AU, et al. (2010) Expanding two-photon intravital microscopy to the infrared by means of optical parametric oscillator. Biophys J. 98(4):715-23.
4. Siffrin V, Brandt AU, Radbruch H, Herz J, et al. (2009) Differential immune cell dynamics in the CNS cause CD4+ T cell compartmentalization. Brain.132(5): 1247-58.
5. Hauser AE, Junt T, Mempel TR, Sneddon MW, et al. (2007) Definition of germinal-center B cell migration in vivo reveals predominant intrazonal circulation patterns. Immunity. 26(5): 655-67.
6. Helmchen F, Denk W. (2005) Deep tissue two-photon microscopy. Nat Methods. 2(12): 932-40.
